# Quality of life in patients with severe mental illness: a cross-sectional survey in an integrated outpatient health care model

**DOI:** 10.1007/s11136-020-02470-0

**Published:** 2020-03-13

**Authors:** Anne Berghöfer, Luise Martin, Sabrina Hense, Stefan Weinmann, Stephanie Roll

**Affiliations:** 1 Institut für Sozialmedizin, Epidemiologie und Gesundheitsökonomie, Charité - Universitätsmedizin Berlin, Luisenstr. 57, 10117 Berlin, Germany; 2 Klinik f. Pädiatrie m.S. Pneumologie, Immunologie und Intensivmedizin, Otto-Heubner-Centrum für Kinder- und Jugendmedizin, Charité - Universitätsmedizin Berlin, Berlin, Germany; 3 Klinik für Psychiatrie, Psychotherapie und Psychosomatik, Vivantes Klinikum Am Urban, Berlin, Germany

**Keywords:** Quality of life, Severe mental disorder, Determinants, Integrated care, WHOQOL-BREF questionnaire, Clinical global impression scale

## Abstract

**Purpose:**

This study (a) assessed quality of life (QoL) in a patient sample with severe mental illness in an integrated psychiatric care (IC) programme in selected regions in Germany, (b) compared QoL among diagnostic groups and (c) identified socio-demographic, psychiatric anamnestic and clinical characteristics associated with QoL.

**Methods:**

This cross-sectional study included severely mentally ill outpatients with substantial impairments in social functioning. Separate dimensions of QoL were assessed with the World Health Organisation’s generic 26-item quality of life (WHOQOL-BREF) instrument. Descriptive analyses and analyses of variance (ANOVAs) were conducted for the overall sample as well as for diagnostic group.

**Results:**

A total of 953 patients fully completed the WHOQOL-BREF questionnaire. QoL in this sample was lower than in the general population (mean 34.1; 95% confidence interval (CI) 32.8 to 35.5), with the lowest QoL in unipolar depression patients (mean 30.5; 95% CI 28.9 to 32.2) and the highest in dementia patients (mean 53.0; 95% CI 47.5 to 58.5). Main psychiatric diagnosis, living situation (alone, partner/relatives, assisted), number of disease episodes, source of income, age and clinical global impression (CGI) scores were identified as potential predictors of QoL, but explained only a small part of the variation.

**Conclusion:**

Aspects of health care that increase QoL despite the presence of a mental disorder are essential for severely mentally ill patients, as complete freedom from the disorder cannot be expected. QoL as a patient-centred outcome should be used as only one component among the recovery measures evaluating treatment outcomes in mental health care.

**Electronic supplementary material:**

The online version of this article (10.1007/s11136-020-02470-0) contains supplementary material, which is available to authorized users.

## Introduction

Quality of life (QoL) has been reported to be reduced in patients with severe mental illnesses. Awareness of the topic in psychiatric research is rising [1, 2]. Because QoL is increasingly being used in studies as a patient-related outcome, the identification and the impact of factors influencing QoL in these patients is of special interest. In addition to the diagnosis, socio-demographic and clinical aspects have been discussed as relevant factors in previous research for specific disease groups [3].

### Affective disorders

Several studies have shown a clinically relevant reduction in QoL in patients with unipolar depression. Socio-demographic factors such as age, sex, relationship status and living situation explained only a small proportion of the variance in QoL [4, 5]. Self-confidence and social support [6] as well as pharmacological [7], cognitive behavioural [8] and psychotherapeutic treatments [9] have been positively associated with QoL in this patient group. Negative associations have been reported with concurrent mental disorders [10] and disease severity [11].

For patients with bipolar depression, scientific reviews have reported a reduced QoL compared to healthy people even in euthymic phases [12, 13]. No association could be found with age, sex or employment status [14]. A negative correlation has been shown for the number of psychopathological symptoms [15], early disease onset [16], and concomitant mental illnesses such as neurotic [17] or addiction [18] disorders. Pharmacological [19] and non-pharmacological treatments [20] have been suggested to improve QoL in bipolar patients. A recent study from Canada reported an increase in mental QoL and decreases in physical QoL under guideline-driven treatment [21].

### Schizophrenia or psychotic disorders

Subjective QoL in patients with schizophrenia is usually lower than in the general population [22, 23] but has been suggested to be higher than in patients with affective disorders [2, 22]. Recent meta-analyses have shown significant moderating effects of socio-demographic factors on QoL in this patient group [23]. Coping with the disease, health-related control beliefs and social support were identified as positive predictors [24], while negative social interactions and the subsequent stigmatisation experienced were identified as negative predictors for QoL [25]. Disease severity [26], disease duration [23], long-term inpatient treatment [27] and adverse reactions to pharmacotherapy [28] were further factors that were negatively correlated with QoL in patients with schizophrenia. In a cohort of older patients with schizophrenia spectrum disorder, depression and cognitive impairment negatively impacted QoL [29].

Studies on QoL in patients with schizoaffective disorder are limited but suggest a reduced QoL compared to the general population [2]. No association has been found with socio-demographic factors [30]; negative associations have been reported with the severity of depressive and negative symptoms as well as physical problems. Social support, self-confidence, and self-efficacy were positively associated with QoL in these patients [31].

### Anxiety disorders

Patients with anxiety disorders have reported a reduced QoL in previous research, especially when diagnosed with a generalised anxiety disorder [32]. Subjective QoL showed no association with socio-demographic factors [33] or disease severity, whereas comorbidity with depression seemed to be negatively associated [34] with QoL. Both pharmacological and psychotherapeutic treatments have been associated with improved QoL in anxiety patients [34–36].

### Alzheimer’s and other dementias

Patients with dementia have reported a subjective QoL level similar to elderly persons without dementia [37, 38]. A literature review on factors influencing QoL in dementia patients has yielded no association with socio-demographic factors [39]. A reduced QoL has been shown in patients with mental health impairments (especially depressive symptoms) [40], a high number of comorbidities [41, 42], pain [43], behavioural problems [44], lack of social support [45] and long-term inpatient care [46]. In a recent meta-analysis, factors reflecting relationships, social engagement and functional ability were associated with a better QoL. Instead, factors indicative of poorer physical and mental health (including depression and other neuropsychiatric symptoms) and poorer carer well-being were associated with poorer QoL [47]. No or only limited associations were observed with pharmacological or non-pharmacological treatments [48, 49]. However, a recent German study showed a stable QoL over time in users of dementia care network services at a level slightly above average, indicating no decrease or worsening over time, as could have been expected [50].

### Alcohol addiction

In patients with alcohol addiction, QoL has been shown to be lower than in the general population [51, 52]. Higher age, female sex, low education, impaired overall health status, level of addiction severity and somatic or psychiatric comorbidities have been identified as potential predictors for reduced QoL [51, 53, 54]. This has been contrasted by an increased QoL in patients who were enrolled in a detoxification programme [55, 56] or received other treatment interventions [57].

### Integrated psychiatric care programme

The present study on QoL was embedded in an integrated psychiatric care (IC) programme for patients with severe mental disorders. Since 2008, this programme has been offered in several federal states within the framework of selective contracts between providers and statutory health insurance funds [58].

The aim of this study was to (a) assess the QoL in patients with severe mental illness participating in an IC programme in Germany, (b) compare the QoL among different diagnostic groups, and (c) identify socio-demographic, psychiatric anamnestic, and clinical characteristics associated with different QoL domains in this population.

## Methods

### Setting and study population

This cross-sectional observational study was performed within a research project at the Charité—Universitätsmedizin Berlin (Germany) for the evaluation of a model of IC. The model was implemented in the regions of Berlin/Brandenburg and Lower Saxony/Bremen to strengthen the network of therapeutic care providers by allowing complex outpatient care for patients with severe mental illnesses [58–61].

The IC model included patients who met the following inclusion criteria:

- aged 18 years or older;
- resided in the participating regions;
- insured with one of the participating statutory health insurances (DAK-Gesundheit, BKK Vertragsarbeitsgemeinschaft Mitte);
- diagnosed with an F0.X to F8.X ICD-10 code;
- needed hospital admission requiring care;
- entitled to receive complex outpatient care instead of inpatient treatment according to the assessment of the attending physician;
- impaired social functioning level (score ≤ 50 on the global assessment of functioning scale (GAF) [62]);
- assessed with illness severity of ≥ 5 on the clinical global impression scale [63] (CGI);
- provided written consent (if individual care support was needed, consent of the caregiver was required as well).

Patients with acute suicidality could not be included in the IC programme.

The present study was based on a subsample of IC patients who were selected between 01 January 2008 and 31 March 2010 and were categorised into seven diagnostic groups based on the following main diagnoses: affective disorders (F30–34, F38, F39), schizophrenia (F20), schizoaffective disorder (F25), neurotic disorders (F40–45, F48), dementia (F00–03), and alcohol-related disorder (F10). The patients with specific personality disorders (F60), other psychoactive substance-related disorders (F19), organic mental disorders other than dementia (F06–07) and other acute or chronic psychotic disorders (F21–24, F29) were excluded from the analysis due to insufficient numbers.

### Outcomes

Patients were asked to complete the World Health Organisation QoL-BREF (WHOQOL-BREF) questionnaire [64, 65]. The WHOQOL-BREF offers a global scale for the overall assessment of QoL that is derived from two separate questions and four constituent domains (physical health, psychological health, social relationships, and environment) with values from 0 to 100 and higher scores indicating a better QoL. The WHOQOL-BREF is regularly used for the assessment of QoL in mentally ill as well as in healthy subjects [66–68]. A definition of a minimal clinically important difference (MCID) on the WHOQOL-BREF has not previously been calculated for mental health patients, although it has been recommended to use the MCID for other diagnoses [69]. Therefore, the proposal by Crocker et al. [70] has been used to define a small difference for the physical, psychological, social, and environmental domains as 3.6, 3.5, 4, and 3.2, respectively.

### Data collection

Patients were consecutively included by the attending physicians. On a quarterly basis, physicians assessed socio-demographic (sex, living situation, source of income, legal care), psychiatric anamnestic (age at disease onset, number of previous psychiatric inpatient stays, overall number of episodes, number of suicide attempts) and clinical data (diagnosis, GAF score, CGI score) according to a standardised manual. The main diagnosis was the one that led to the acute need for psychiatric therapy and admission to the IC. The patients completed the WHOQOL-BREF at inclusion in the IC and at the beginning of every quarter year. The physicians’ documentations and the patients’ questionnaires were checked by trained study personnel according to standard operating procedures (SOPs), and continuous quality circles with the participating physicians were implemented [58].

### Statistical analysis

The present analysis includes only cross-sectional data collected at the time of inclusion in the IC. Statistical analysis was conducted with SPSS 19.0 for Microsoft Windows. Socio-demographic, psychiatric anamnestic and clinical patient characteristics were described for the entire IC sample and stratified by main diagnostic group by mean and standard deviation (SD) or frequencies and percentages.

In the first step, the association between age, sex and main diagnosis was assessed for each of the diagnostic groups of interest to identify potentially confounding factors. In a second step, QoL mean values were compared among diagnostic groups separately for each WHOQOL-BREF domain and for the overall QoL scale using analysis of variance (ANOVA). The reference values for QoL of the German general population were taken from Angermeyer et al. [68].

Due to differences in the QoL values among the diagnostic groups, means were adjusted for diagnosis but not for age and sex (no relevant differences). To identify the influence of patient characteristics on the QoL, adjusted mean values for each of the WHOQOL-BREF domains and the overall QoL scale are presented by socio-demographic, psychiatric anamnestic and clinical characteristics, based on domain-specific models of multivariable ANOVA (with R^2^ and adjusted R^2^ values). Results were checked for multicollinearity and heteroscedasticity. All results are considered exploratory (without any formal significance level).

Ethical approval was obtained from the Ethics Committee of the Charité—Universitätsmedizin Berlin (EA1/088/08).

## Results

Overall, 1433 patients were included in the IC programme, of which 1347 patients had one of the study diagnoses. Among those, 953 completed the WHOQOL-BREF questionnaire. The calculation of scores for the subdomains of the WHOQOL-BREF was possible for all 953 patients, while the assessment of the global score was possible for 941 patients.

The largest groups were patients with unipolar depression (58.1%) and psychotic disorders (13.7%), followed by neurotic disorders (11.7%) (Table 1). Approximately three-quarters of the neurotic patients suffered from an anxiety disorder (data not shown). Dementia was diagnosed in 5.2% of patients, of which approximately 70% were affected by Alzheimer’s disease. The mean age in the overall sample was 47.4 years. More patients were female (69.3%), and a large part received pension (old age and disability), social welfare or unemployment/sickness benefits. Half of the patients were living alone at the time of assessment.

**Table 1:** Socio-demographic characteristics of severely mentally ill patients included in an integrated care programme, stratified by diagnostic group

	Overall	Unipolar depression	Bipolar depression	Schizophrenia	Schizoaffective disorder	Neurotic disorder	Dementia	Alcohol addiction
Number of patients (*n*, %)	953	554 (58.1)	49 (5.1)	131 (13.7)	45 (4.7)	109 (11.4)	50 (5.2)	15 (1.6)
Sex (*n* = 953) (*n*, %)								
Male	293 (30.7)	154 (27.8)	18 (36.7)	53 (40.5)	9 (20.0)	33 (30.3)	18 (36.0)	8 (53.3)
Female	660 (69.3)	400 (72.2)	31 (63.3)	78 (59.5)	36 (80.0)	76 (69.7)	32 (64.0)	7 (46.7)
Age in years (*n* = 953)								
(Mean ± SD)	47.4 ± 15.8	46.3 ± 14.9	49.8 ± 13.0	41.4 ± 12.9	46.8 ± 14.4	45.4 ± 12.9	77.9 ± 6.9	49.5 ± 11.0
(Median, range)	46 (17–88)	46 (17–87)	50 (22–76)	42 (17–74)	46 (21–78)	45 (19–80)	78 (55–88)	47 (34–68)
Source of income (*n* = 951) (*n*, %)								
Own income	182 (19.1)	122 (22.1)	11 (22.4)	11 (8.5)	5 (11.1)	31 (28.4)	0 (0)	2 (13.3)
Pension	340 (35.8)	151 (27.3)	23 (46.9)	56 (43.1)	27 (60.0)	26 (23.9)	49 (98.0)	8 (53.3)
Unemployment or sickness benefit	171 (18.0)	128 (23.1)	5 (10.2)	8 (6.2)	4 (8.9)	24 (22.0)	0 (0)	2 (13.3)
Social welfare	178 (18.4)	101 (18.3)	6 (12.2)	44 (33.8)	6 (13.3)	19 (17.4)	0 (0)	2 (13.3)
Support by caregiver	80 (8.4)	51 (9.2)	4 (8.2)	11 (8.5)	3 (6.7)	9 (8.3)	1 (2.0)	1 (6.7)
Living (*n* = 950) (*n*, %)								
Alone	471 (49.7)	246 (44.6)	33 (67.3)	83 (63.8)	28 (62.2)	60 (56.1)	13 (26.0)	8 (57.1)
With partner or relative	409 (43.2)	270 (48.9)	15 (30.6)	33 (25.4)	14 (31.1)	42 (39.3)	29 (58.0)	6 (42.9)
In parents’ house	47 (5.0)	28 (5.1)	1 (2.0)	11 (8.5)	3 (6.7)	4 (3.7)	0 (0)	0 (0)
Assisted living	20 (2.1)	8 (1.4)	0 (0)	3 (2.3)	0 (0)	1 (0.9)	8 (16.0)	0 (0)
Care situation (*n* = 950) (*n*, %)								
Legal care	102 (10.7)	27 (4.9)	9 (18.4)	39 (29.8)	8 (17.8)	6 (5.6)	10 (20.0)	3 (20.0)
No legal care	848 (89.3)	525 (94.8)	40 (81.6)	92 (70.2)	37 (82.2)	102 (94.4)	40 (80.0)	12 (80.0)

Approximately half of the patients had at least one co-occurring psychiatric diagnosis (Table 2). The social functioning level based on the mean GAF was 36.6, which indicated a strong social impairment in multiple areas. Approximately 40% of the patients were defined as extremely/most extremely ill according to the CGI scale.

**Table 2:** Psychiatric anamnestic and clinical characteristics of severely mentally ill patients included in an integrated care programme, stratified by main diagnosis (index episode = episode that caused inclusion in the study)

	Overall	Unipolar depression	Bipolar depression	Schizophrenia	Schizoaffective disorder	Neurotic disorder	Dementia	Alcohol addiction
Disease duration in years (*n* = 740)								
(Mean ± SD)	10.5 ± 11.0	9.4 ± 10.8	15.7 ± 12.5	13.8 ± 11.0	14.5 ± 9.8	11.2 ± 11.0	2.8 ± 2.3	17.5 ± 13.9
Number of episodes, including index episode (*n* = 515)								
(mean ± SD)	4.5 ± 6.5	3.6 ± 4.4	7.3 ± 6.1	5.8 ± 5.6	6.7 ± 7.3	5.3 ± 14.1	1.3 ± 0.7	6.3 ± 4.2
1 episode (*n*, %)	146 (28.3)	100 (32.6)	3 (9.4)	10 (12.2)	4 (14.3)	20 (40.0)	8 (80.0)	1 (16.7)
2 episodes (*n*, %)	82 (15.9)	63 (20.5)	0 (0)	9 (11.0)	0 (0)	8 (16.0)	1 (10.0)	1 (16.7)
3 episodes (*n*, %)	99 (19.2)	61 (19.9)	6 (18.8)	22 (26.8)	3 (10.7)	6 (12.0)	1 (10.0)	0 (0)
4–9 episodes (*n*, %)	118 (22.9)	54 (17.6)	15 (46.9)	23 (28.0)	15 (53.6)	10 (20.0)	0 (0)	1 (16.7)
10 or more episodes (*n*, %)	70 (13.6)	29 (9.4)	8 (25.0)	18 (22.0)	6 (21.4)	6 (12.0)	0 (0)	3 (50.0)
Number of previous psychiatric inpatient stays (*n* = 363)								
(mean ± SD)	2.9 ± 4.4	1.8 ± 2.7	4.5 ± 4.9	5.5 ± 6.6	5.7 ± 4.7	2.5 ± 4.8	0.6 ± 0.8	6.0 ± 3.7
None	114 (31.4)	82 (40.4)	5 (20.8)	5 (8.3)	2 (8.7)	15 (36.6)	4 (57.1)	1 (20.0)
1 episode (*n*, %)	60 (16.5)	35 (17.2)	2 (8.3)	11 (18.3)	1 (4.3)	9 (22.0)	2 (28.6)	0 (0)
2 episodes (*n*, %)	57 (15.7)	35 (17.2)	2 (8.3)	8 (13.3)	3 (13.0)	8 (19.5)	1 (14.3)	0 (0)
3–6 episodes (*n*, %)	90 (24.8)	43 (21.2)	10 (41.7)	20 (33.3)	9 (39.1)	6 (14.6)	0 (0)	2 (40.0)
7 or more episodes (*n*, %)	42 (11.6)	8 (3.9)	5 (20.8)	16 (26.7)	8 (34.8)	3 (7.3)	0 (0)	2 (40.0)
Number of suicide attempts (*n* = 650)								
(mean ± SD)	0.6 ± 1.3	0.6 ± 1.3	0.6 ± 1.1	0.8 ± 1.1	0.8 ± 1.6	0.5 ± 1.2	0.1 ± 0.3	1.4 ± 1.7
None	455 (70.0)	279 (71.5)	31 (72.1)	50 (56.2)	17 (68.0)	60 (75.9)	14 (93.3)	4 (44.4)
1 (*n*, %)	101 (15.5)	61 (15.6)	3 (7.0)	20 (22.5)	4 (16.0)	11 (13.9)	1 (6.7)	1 (11.1)
2 or more (*n*, %)	94 (14.5)	50 (12.8)	9 (20.9)	19 (21.3)	4 (16.0)	8 (10.1)	0 (0)	4 (44.4)
Co-occurring psychiatric diagnosis (*n* = 953) (*n*, %)								
None	451 (47.3)	257 (46.4)	28 (57.1)	85 (64.9)	29 (64.4)	18 (16.5)	34 (68.0)	0 (0)
1	291 (30.5)	167 (30.1)	15 (30.6)	31 (23.7)	11 (24.4)	47 (43.1)	13 (26.0)	7 (46.7)
2 or more	211 (22.1)	130 (23.5)	6 (12.2)	15 (11.5)	5 (11.1)	44 (40.4)	3 (6.0)	8 (53.3)
Co-occurring somatic diagnosis (*n* = 953) (*n*, %)								
None	733 (76.9)	432 (78.0)	41 (83.7)	111 (84.7)	35 (77.8)	80 (73.4)	22 (44.0)	12 (80.0)
1 or more	220 (23.1)	122 (22.0)	8 (16.3)	20 (15.3)	10 (22.2)	29 (26.6)	28 (56.0)	3 (20.0)
GAF score (*n* = 943)								
(Score, mean ± SD)	36.6 ± 8.1	37.2 ± 7.9	37.8 ± 8.2	35.8 ± 7.3	35.8 ± 7.8	38.0 ± 8.5	29.2 ± 8.4	33.7 ± 9.0
CGI severity (*n* = 943)								
(Mean ± SD)	5.4 ± 0.7	5.4 ± 0.6	5.4 ± 0.5	5.4 ± 0.8	5.4 ± 0.7	5.3 ± 0.7	5.4 ± 0.7	5.8 ± 0.7
Moderately or markedly ill (CGI = 4 or 5) (*n*, %)	586 (61.9)	339 (61.5)	29 (59.2)	78 (60.9)	27 (60.0)	74 (67.9)	34 (69.4)	5 (33.3)
Extremely or most extremely ill (CGI = 6 or 7) (*n*, %)	360 (38.1)	212 (38.5)	20 (40.8)	50 (39.1)	18 (40.0)	35 (32.1)	15 (30.6)	10 (66.7)

*CGI* clinical global impression, *GAF* global assessment of functioning

**Fig. 1:**
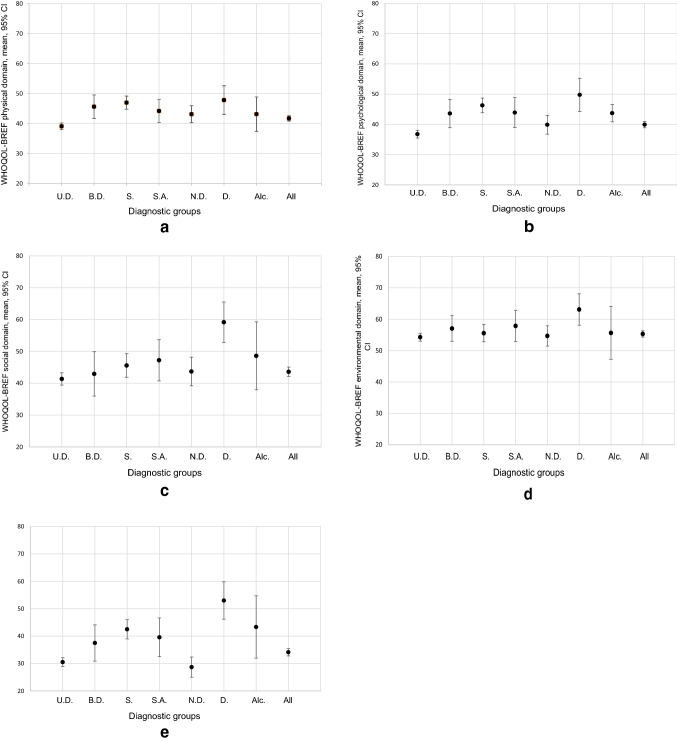
**a**–**e** Mean quality of life (QoL) with 95% CI by main diagnosis in each of the domains. The line indicates the mean value of the respective WHOQOL-BREF domain in the general German population [68]. *U.D*. unipolar depression, *B.D*. bipolar depression, *S*. schizophrenia, *S.A*. schizoaffective disorder, *N.D*. neurotic disorder, *D*. dementia, *Alc*. alcohol addiction

QoL was generally low in all patients (Supplemental Table S1; Fig. 1a–e). The lowest global scale score was reported for patients with neurotic disorders, while in each of the domains, QoL was lowest in patients with unipolar depression. As the inclusion of age and sex into the analysis models did not lead to relevant changes in the estimates, unadjusted QoL mean values are presented. Additionally, tests for the interaction between age, sex and main diagnosis showed no relevant results.

QoL was lower in all diagnostic groups than in the general population [68] for all WHOQOL-BREF domains (Fig. 1a–e).

In the physical health domain, the unipolar depressive patients reported a lower QoL than all other diagnostic groups. Clinically relevant differences were observed between the patients with unipolar depression (39.1; 95% CI 38.0 to 40.2) and those with bipolar depression (45.6; 95% CI 41.7 to 49.5), schizophrenia (47.0; 95% CI 44.8 to 49.2) and dementia (47.8; 95% CI 43.0 to 52.6). Psychological health-related QoL was impaired mainly in the patients with unipolar depression and with neurotic disorders, who showed markedly lower values than the patients with schizophrenia and dementia. In the domain of social relationships, the patients with dementia showed a notably higher QoL level than the patients in four out of the remaining six diagnostic groups. Regarding the environmental domain, QoL was higher in the dementia patients than in the patients with unipolar depression and those with neurotic disorders. Additionally, on the WHOQOL-BREF global scale, the patients with dementia were the least impaired group, which was shown by higher values than most of the other diagnostic groups (except for alcohol addiction). Furthermore, the schizophrenia patients reported a markedly higher QoL than those with unipolar depression or neurotic disorders.

To estimate the association between socio-demographic, psychiatric anamnestic and clinical factors and QoL, domain-specific multivariate models were fit. All potential confounding socio-demographic, psychiatric anamnestic and clinical factors (see Tables 1, 2) were included with the exception of the number of hospital stays and the disease duration that highly correlated with the number of episodes. Additionally, somatic co-diagnoses were not included based on the findings of low validity of documented somatic co-diagnoses in this study [71].

The largest adjusted mean differences were observed among the main diagnostic groups in the global scale model (see Table 3). QoL values ranged from 32.2 (95% CI 24.2 to 40.3) in the patients with unipolar depression to 55.0 (95% CI 34.1 to 75.9) in the patients with alcohol addiction. To a lesser extent, this pattern was present in the physical, psychological and environmental domains. In the social domain, the highest QoL was reported in the schizoaffective disorder patients. The differences were larger than a small difference as defined by Crocker et al. [70] in each of the models. The CIs were, however, overlapping in all models, and wide CIs indicated a generally limited precision of the estimates. Further notable differences were identified for living situation with the highest QoL for assisted living in all except for the psychological domain, where the lowest QoL was reported by the people living in assisted housing (38.7; 95% CI 22.7 to 54.6). Less pronounced but still clinically relevant differences were shown for source of income, where the widest range was observed in the social domain (social welfare: 42.7; 95% CI 32.3 to 53.1 vs. own income: 54.4; 95% CI 44.2 to 64.6), without, however, showing a clear QoL pattern among characteristics of the variable. Sex and psychiatric co-diagnosis yielded no relevant QoL differences. In the psychological domain, a higher age indicated a slightly higher QoL (*r* = 0.17; 95% CI 0.03 to 0.31), while a high CGI score was associated (fulfilling the criteria of statistical relevance with the 95% CIs not containing the null value) with a lower QoL in the global scale section (*r* =  − 4.07; 95% CI − 7.35 to − 0.78) and the environmental domain (*r* =  − 3.10; 95% CI − 5.67 to − 0.54).

**Table 3:** Associations between socio-demographic, psychiatric anamnestic and clinical factors and quality of life by WHOQOL-BREF sections based on domain-specific multifactorial analyses of variance

	WHOQOL-BREF	WHOQOL-BREF domains			
	Global Scale	Physical	Psychological	Social	Environmental
	Mean (95% CI)	Mean (95% CI)	Mean (95% CI)	Mean (95% CI)	Mean (95% CI)
Main diagnosis (*F*, df)	(3.30, 6)	(2.36, 6)	(4.93, 6)	(0.99, 6)	(0.73, 6)
Unipolar depression	32.2 (24.2 to 40.3)	40.1 (34.3 to 45.8)	34.7 (28.3 to 41.0)	44.1 (35.0 to 53.2)	52.5 (46.3 to 58.8)
Bipolar depression	43.5 (32.7 to 54.2)	47.3 (39.6 to 55.1)	44.8 (36.3 to 53.3)	48.5 (36.3 to 60.7)	54.2 (45.8 to 62.6)
Schizophrenia	40.5 (30.9 to 50.0)	46.4 (39.5 to 53.2)	45.2 (37.7 to 52.7)	50.0 (39.2 to 60.8)	53.5 (46.0 to 60.9)
Schizoaffective disorder	39.4 (27.5 to 51.3)	43.1 (34.5 to 51.6)	40.0 (30.6 to 49.5)	53.6 (40.0 to 67.1)	56.1 (46.8 to 65.4)
Neurotic disorder	32.4 (23.2 to 41.6)	44.1 (37.5 to 50.7)	41.1 (33.8 to 48.2)	46.9 (36.4 to 57.3)	53.7 (46.5 to 60.9)
Dementia	45.9 (30.1 to 61.6)	43.4 (32.1 to 54.7)	39.6 (27.2 to 52.1)	50.3 (32.4 to 68.2)	58.0 (45.7 to 70.4)
Alcohol addiction	55.0 (34.1 to 75.9)	48.1 (32.1 to 54.7)	50.7 (34.2 to 67.3)	49.7 (25.9 to 73.5)	66.3 (49.9 to 82.6)
Sex (*F*, df)	(0.35, 1)	(1.98, 1)	(0.06, 1)	(0.22, 1)	(0.07, 1)
Male	41.9 (33.5 to 50.4)	45.8 (39.7 to 51.8)	42.5 (35.9 to 49.2)	49.6 (40.0 to 59.2)	56.6 (50.0 to 63.2)
Female	40.6 (32.3 to 48.8)	43.5 (37.6 to 49.4)	42.1 (35.6 to 48.6)	48.4 (39.0 to 57.8)	56.1 (49.6 to 62.5)
Living situation (*F*, df)	(0.13, 5)	(1.06, 5)	(1.26, 5)	(0.64, 5)	(0.95, 5)
Living alone	41.5 (36.7 to 46.3)	45.5 (42.0 to 48.9)	45.8 (42.0 to 49.6)	46.0 (40.5 to 51.4)	57.5 (53.8 to 61.3)
With partner or relatives	41.4 (36.3 to 46.5)	43.7 (40.0 to 47.4)	43.6 (39.6 to 47.7)	49.2 (43.4 to 55.0)	60.2 (56.2 to 64.2)
With parents	40.5 (30.9 to 50.1)	46.3 (39.4 to 53.2)	50.9 (43.3 to 58.4)	46.1 (35.2 to 56.9)	61.1 (53.6 to 68.5)
Assisted living	46.4 (26.2 to 66.6)	56.2 (41.7 to 70.7)	38.7 (22.7 to 54.6)	60.5 (37.5 to 83.4)	60.5 (44.8 to 76.3)
Subsistence (*F*, df)	(0.62, 4)	(0.76, 4)	(2.24, 4)	(2.97, 4)	(2.31, 4)
Own income	42.5 (33.5 to 51.5)	45.5 (39.1 to 52.0)	43.8 (36.7 to 50.9)	54.4 (44.2 to 64.6)	58.2 (51.2 to 65.3)
Retirement/disability pension	37.8 (29.3 to 46.4)	42.0 (35.9 to 48.2)	37.7 (30.9 to 44.5)	48.1 (38.4 to 57.9)	55.9 (49.2 to 62.6)
Unemployment/sick benefit	41.0 (32.0 to 50.1)	45.9 (39.4 to 52.4)	45.3 (38.1 to 52.4)	52.0 (41.7 to 62.3)	58.6 (51.5 to 65.7)
Social welfare	42.7 (33.6 to 51.9)	45.1 (38.5 to 51.7)	42.9 (35.7 to 50.2)	42.7 (32.3 to 53.1)	51.7 (44.6 to 59.0)
Support by partners/family	42.2 (31.7 to 52.7)	44.6 (37.1 to 52.1)	41.9 (33.6 to 50.2)	47.7 (35.8 to 59.6)	57.2 (49.0 to 65.4)
Psychiatric co-diagnoses (*F*, df)	(1.04, 2)	(2.50, 2)	(0.23, 2)	(0.29, 2)	(0.64, 2)
None	42.7 (34.4 to 51.0)	44.5 (38.5 to 50.4)	42.9 (36.3 to 49.4)	48.1 (38.7 to 57.6)	55.9 (49.4 to 62.4)
One	42.3 (33.6 to 51.0)	47.0 (40.8 to 53.2)	42.7 (35.8 to 49.5)	50.1 (40.3 to 60.0)	57.6 (50.9 to 64.4)
More than one	38.9 (30.0 to 47.6)	42.5 (36.1 to 48.8)	41.4 (34.4 to 48.4)	48.7 (38.7 to 58.8)	55.4 (48.5 to 62.3)

	WHOQOL-BREF	WHOQOL-BREF domains			
	Global Scale	Physical	Psychological	Social	Environmental
	Regr. coeff. (95% CI)	Regr. coeff. (95% CI)	Regr. coeff. (95% CI)	Regr. coeff. (95% CI)	Regr. coeff. (95% CI)
Age (year) (*F*, df)	(0.14, 1) 0.03 (− 0.15 to 0.21)	(0.67, 1) 0.05 (− 0.07 to 0.18)	(5.38, 1) 0.17 (0.03 to 0.31)	(0.00, 1) − 0.01 (− 0.21 to 0.20)	(1.09, 1) 0.07 (− 0.07 to 0.21)
Number of episodes (*F*, df)	(0.25, 1) 0.08 (− 0.23 to 0.39)	(1.20, 1) 0.12 (− 0.10 to 0.35)	(0.52, 1) 0.09 (− 0.15 to 0.33)	(0.00, 1) − 0.00 (− 0.35 to 0.35)	(0.32, 1) 0.07 (− 1.7 to 0.31)
Number of suicide attempts (*F*, df)	(1.03, 1) 0.83 (− 0.79 to 2.46)	(0.20, 1) − 0.26 (− 1.43 to 0.90)	(1.22, 1) − 0.72 (− 2.01 to 0.56)	(0.40, 1) 0.59 (− 1.25 to 2.44)	(0.04, 1) 0.14 (− 1.13 to 1.4)
GAF score (*F*, df)	(0.23, 1) 0.07 (− 0.20 to 0.33)	(0.32, 1) 0.06 (− 0.14 to 0.25)	(0.10, 1) 0.03 (− 0.18 to 0.25)	(1.31, 1) − 0.18 (− 0.48 to 0.13)	(0.00, 1) 0.01 (− 0.20 to 0.22)
CGI severity score (*F*, df)	(5.92, 1) − 4.07 (− 7.35 to − 0.78)	(2.71, 1) − 1.97 (− 4.33 to 0.38)	(2.99, 1) − 2.28 (− 4.88 to 0.31)	(0.11, 1) 0.64 (− 3.09 to 4.36)	(5.65, 1) − 3.10 (− 5.67 to − 0.54)
*R*^2^/corrected *R*^2^	0.084/0.029	0.088/0.035	0.116/0.064	0.056/0.001	0.067/0.012

The results are expressed as the means with 95% confidence intervals (CIs) for categorical variables or regression coefficients with 95% CI for continuous variables (all results adjusted for main diagnosis, living situation, subsistence, sex, psychiatric co-diagnoses, age, number of episodes, number of suicidal attempts, GAF score, and CGI score)

*GAF* global assessment of function, *CGI* clinical global impression

## Discussion

Overall, QoL in the clinical sample on which this study was based was lower than the QoL in the general population for the global score as well as for all four WHOQOL-BREF domains, which is in line with other research [72]. The lowest impairment was reported in the environmental domain. The assessment of QoL by diagnostic group showed diagnosis-specific differences. As also shown in previous studies [73–75], the lowest QoL levels were found in the patients with unipolar depression or neurotic disorders in contrast to dementia patients who reported by far the highest WHOQOL-BREF global score levels. QoL is a subjective measure that is not necessarily reduced in severe diseases (such as schizophrenia) unlike mental disorders such as unipolar depression. In the case of schizophrenia, our study found similar results as Franz et al. [76] underscoring the hypothesis that patients with psychotic illness may compare themselves predominantly laterally or downwards relative to fellow patients and not with the general population.

Relevant differences in QoL levels were observed among the main psychiatric diagnostic groups, living situation and source of income categories. In some domains, age and the clinical global impression based on the CGI scale were identified as potential predictors of QoL in this sample of integrated care patients. Interestingly, the level of social functioning was no predictor of QoL. Overall, only a small part of the variation in the global score as well as for WHOQOL-BREF domains could be explained by socio-demographic, psychiatric anamnestic or clinical patient characteristics, which was in line with previous findings [4, 6, 55, 77]. The explained variation in the WHOQOL-BREF section-specific models approximately corresponded to results that were published by Trompenaars et al. [5] who had assessed the influence of demographic patient characteristics on WHOQOL-BREF domains in a psychiatric outpatient sample.

Regarding the living situation, the highest QoL was found in patients living in an assisted home in most of the domains. This suggests that living in an assisted home incorporates social support and integration in a protected living situation, thereby positively influencing the patient’s QoL [67]. In a recent study from China in unipolar depressive patients, being married yielded a positive impact on all QoL domains [78], which was not supported by our findings. Cultural diversities as well as differences in the study populations did, however, allow only very limited comparability in this regard. The positive effect of paid work on the QoL of psychiatric patients has been described in detail in the relevant literature [67, 79, 80]. Having one’s own income might be associated with a better economic situation and higher financial security or autonomy. In addition, having one's own income is likely to be related to having a job, which has been related not only to financial advantages but also to the benefits of having a social network at work [81]. However, no clear pattern in the present study could be observed in this regard.

A lack of or only a small association of sex and age on QoL in mentally ill populations has been found in our study as well as in several other studies and meta-analyses [82, 83]. However, a Dutch study on QoL in psychiatric ambulatory care patients reported a higher QoL in women in the social relationships domain [67] and a decreasing QoL with increasing age in the social relationship and the physical health domains [84].

The number of disease episodes did not have a relevant influence on any of the QoL domains. However, in previous studies, severely ill patients have reported seemingly paradoxical positive QoL, which might be explained by a so-called response shift bias [85, 86].

Previous studies have often reported an association between psychiatric comorbidities and reduced QoL in different patient groups [87–89]. In the present study, the highly significant negative association between QoL and co-occurring psychiatric disorders did not persist in the final models. However, the tendency of a decreasing QoL with an increasing number of psychiatric diagnoses can be observed in some of the QoL domains.

Furthermore, some studies have shown an association between suicidality and QoL in patients with affective disorders [90] or schizophrenia [87], which was not supported by the present results. The lack of association between the WHOQOL-BREF domains and disease duration was in line with a study by Nordt et al. [91], who did not find a relationship between QoL in severely mentally ill patients and the time of disease onset.

An association was identified between disease severity and QoL in the global and environmental domains. A worse overall clinical impression (higher CGI value) was related to a lower QoL in these domains, which was also observed by Henkel et al. [92].

Independent of socio-demographic, psychiatric anamnestic, and clinical characteristics, the main diagnostic group remained the factor with the most distinct differences in all models. This suggests QoL as a diagnosis-specific aspect that should be taken into account in evaluation studies in different patient groups.

Overall, these exploratory results indicate potential clinical relevance of some of the factors investigated in the present study. They should, however, be cautiously interpreted against the background of a limited precision due to a small number of cases in some categories, which resulted in rather large CIs. The selection of factors potentially influencing the patients’ QoL in this study was limited to variables that were assessed in the context of the research project for the IC model evaluation. Therefore, factors that had been identified as potential determinants in other studies, such as self-esteem, individual expectations, personality traits, self-efficacy [93], illness insight [94], self-stigma [95], or pharmacotherapeutic side effects [94], were not considered. Also, the findings might have been biased by (time constant) unobserved confounders. Further factors than those taken into account, such as the treatment setting or the possibility of participation in therapeutic decisions, should be considered as relevant predictors for QoL in psychiatric patients in future studies.

The statistical examination for multicollinearity revealed indications of dependencies between a few of the variables (e.g. living situation and subsistence). However, we chose to keep all of the factors in our analyses. One reason is that collinearity mostly is a concern to result in essential shifts in the *p* values of one predictor variable (i.e. reduction in power) when another predictor is included in the model. However, since this was an exploratory study without adjustments for multiple testing, *p* values were not the focus of our analyses. In addition, dropping important variables from the model might have introduced bias. In our view, our data might not be suitable for disentangling all of the combination effects which are common in socio-demographic and lifestyle factors. A graphical check did not find relevant evidence for heteroscedasticity. Hence, no methods to account for heteroscedasticity (e.g. robust standard errors) were used.

Despite the controversial discussion about the assessment of QoL in psychiatric patients [96], the concept is increasingly being used in addition to clinical outcomes in mentally ill patients, and the evidence suggests that the WHOQOL-BREF is a valid instrument in this context [40, 84, 97].

The interpretation of the results is hampered by the lack of a definition of MCID of different QoL measures as patient-reported outcomes. The MCID is the smallest difference in a score that a patient would identify as important. In our study, no control group was available neither did we test the effects of an intervention. Responsiveness of the WHOQOL-BREF instrument has been shown in a variety of settings and conditions. The instrument is able to detect even small changes induced by treatments as shown by effect sizes in more than 20 studies being highly significant even with low or moderate values (Cohen’s *d* between 0.10 and 0.37) [98]. However, the clinical meaning of WHOQOL-BREF differences in psychiatric patients is still under-researched. While it is well known that depressive symptomatology, independent of clinical psychiatric diagnoses, affects patients` quality-of-life judgement [99, 100], there are no available studies investigating MCIDs in people with different psychiatric diagnoses. One recent review which assessed the usability of MCIDs for measuring meaningful changes in disease-specific and generic health-related quality-of-life outcomes found only two studies in mental health with patients with schizophrenia; one study used the Heinrichs–Carpenter QoL and the other study used the Lenert Positive and Negative Syndrome Scale, PANSS [101]. In the absence of valid evidence regarding established MCID across different generic and disease-specific quality-of-life measures and taking into account that the MCID is context specific and not a fixed attribute [102], we used age- and sex-specific standard values from the WHOQOL-BREF test manual and confidence intervals including *F* scores and df to describe differences between people with varying diagnoses and clinical as well as socio-demographic characteristics [68].

The study population exclusively consisted of outpatient and mostly female, unipolar depressive patients from selected regions in Germany that were enrolled in an IC project, which limits generalisation of the results. In addition, due to a low number of patients in some of the diagnostic groups, differentiation into clinically relevant subgroups was not possible; e.g. ‘dementia’ included patients with different forms of dementia (mainly Alzheimer’s disease), while ‘neurotic disorders’ comprised mainly but not only anxiety patients.

It also needs to be considered that analyses were performed only for patients who completely answered the WHOQOL-BREF, which might selectively exclude more severely ill patients. Especially for dementia patients, non-declared support by family members for filling in the questionnaire cannot be excluded. Additionally, physicians might have graded patients as more severely ill on the psychopathological scales to allow for the patients’ inclusion in the IC programme. Because no causal conclusions can be drawn due to the cross-sectional study design, further prospective longitudinal studies would be desirable.

A notable strength of the study was that it allowed an analysis and a comparison of the QoL among different diagnostic groups based on a large psychiatric outpatient sample. It thereby differs from studies conducted in inpatient settings and provides results that are highly transferable to clinical practice and care.

In summary, the results provide further indication that socio-demographic and clinical variables have little impact on the QoL of people with mental illness. However, if factors that can be improved by psychiatric or psychosocial interventions have only a limited influence on QoL in the short to medium term, the question arises whether QoL is an appropriate outcome in mental health care research. Severely mentally ill patients are often affected by recurrent disease episodes or chronic disease courses, and only a minority can expect to stay completely free from symptoms for the remainder of their life. Especially for those patients, health care that improves QoL despite the presence of the illness is essential.

A recent review emphasised that there is an association between low social functioning and negative QoL in psychotic disorders [103]. The results indicated that factors such as social integration, mobility and adjustment may be more relevant for QoL than mere illness factors. The newly developed instrument “Recovering Quality of Life” (ReQoL) seeks to integrate themes such as hope, relationship, self-perception, and autonomy into established items such as activity, physical health, and well-being to evaluate QoL in patients with mental health [104, 105].

Scepticism about the increasingly widespread use of QoL as an outcome in psychiatric health care research was expressed more than 10 years ago [76, 106], against the background that the assessment of QoL has become routine in many areas of research. However, in recent years, the use of this outcome has continued to increase. Since 2005, publications in Medline under the MeSH terms “quality of life” and “mental health” have more than tripled.

QoL is a markedly subjective measure, and the individual rating depends on the underlying type of mental disorder. QoL can also be seen within a personal frame of reference. This frame of reference is formed by the level of social functioning and the degree of integration into society. When estimating QoL values, this subjective frame of reference must be taken into account. If the change in QoL is used as an outcome after an intervention or after an observation period, the typical characteristics such as measurability, change sensitivity, reliability and validity must also be considered.

As a conclusion of the study, symptoms of mental disorder, clinical impression, and social functioning alone are not sufficient as outcome measures because they do not reflect the subjective patient’s perspective. However, QoL as a patient-centred outcome measure is not unproblematic either. Therefore, the effects of health care should not be measured by the change in QoL alone, but QoL should only be used as one component alongside other recovery measures. Future research should look for alternative patient-related outcomes that better reflect the success of long-term psychiatric care.

## Electronic supplementary material

Below is the link to the electronic supplementary material.

Supplementary file1 (PDF 22 kb)
