# Supplementary material for: Quality of life in patients with severe mental illness: a cross-sectional survey in an integrated outpatient health care model
Source: Qual Life Res. 2020 Mar 13;29(8):2073–87. doi: 10.1007/s11136-020-02470-0 (PMC7363717; doi:10.1007/s11136-020-02470-0)
Supplement: Supplementary file 1 — Supplementary file1 (PDF 22 kb) [file 11136_2020_2470_MOESM1_ESM.pdf]

Supplementary Material

Table S1 Quality of life (WHOQOL-BREF) overall and stratified by diagnostic group (means with 95%-confidence intervals (CI))

	WHOQOL-BREF				
		Domains			
	Global scale	Physical	Psychological	Social	Environmental
**Overall** *(n=953 for subdomains, n=941 for global scale)*	34.1 *[32.8-35.5]*	41.7 *[40.8-42.6]*	39.9 *[40.9-38.9]*	43.6 *[42.1-45.1]*	55.3 *[54.3-56.3]*
**Unipolar depression** *(n=554)*	30.5 *[28.9-32.2]*	39.1 *[38.0-40.2]*	36.8 *[35.5-38,1]*	41.4 *[39.4-43.3]*	54.3 *[53.0-55.5]*
**Bipolar depression** *(n=48)*	37.5 *[31.9-43.1]*	45.6 *[41.7-49.5]*	43.6 *[38.9-48.3]*	42.9 *[36.0-49.9]*	57.0 *[52.9-61.2]*
**Schizophrenia** *(n=131)*	42.5 *[39.1-45.9]*	47.0 *[44.8-49.2]*	46.3 *[43.9-48.7]*	45.6 *[41.9-49.3]*	55.6 *[52.8-58.3]*
**Schizoaffective disorder** *(n=45)*	39.6 *[33.6-45.6]*	44.2 *[40.3-48.1]*	43.9 *[38.9-48.8]*	47.2 *[40.7-53.7]*	57.9 *[52.9-62.8]*
**Neurotic disorder** *(n=109)*	28.7 *[24.9-32.5]*	43.1 *[40.3-45.9]*	39.9 *[36.8-43.0]*	43.7 *[39.2-48.2]*	54.7 *[51.5-57.9]*
**Dementia** *(n=50)*	53.0 *[47.5-58.5]*	47.8 *[43.0-52.6]*	49.8 *[44.3-55.3]*	59.2 *[52.8-65.5]*	63.1 *[58.1-68.1]*
**Alcohol addiction** *(n=15)*	43.3 *[33.3-53.4]*	43.1 *[37.4-48.9]*	43.7 *[40.8-46.6]*	48.6 *[37.9-59.3]*	55.6 *[47.2-64.1]*
